# Leveraging artificial intelligence for inclusive maternity care: Enhancing access for mothers with disabilities in Africa

**DOI:** 10.1177/17455057251326675

**Published:** 2025-03-16

**Authors:** Obasanjo Bolarinwa, Aliu Mohammed, Victor Igharo, Sinegugu Shongwe

**Affiliations:** 1 Department of Public Health, York St John University, London, UK; 2 Demography and Population Studies Programme, Schools of Public Health and Social Sciences, University of the Witwatersrand, Johannesburg, South Africa; 3 Department of Health, Physical Education and Recreation, University of Cape Coast, Cape Coast, Ghana; 4 William H. Gates Institute for Population and Reproductive Health, Johns Hopkins Bloomberg School of Public Health, Baltimore, USA; 5 Department of Public Health Medicine, University of KwaZulu-Natal, Durban, South Africa

**Keywords:** artificial intelligence, maternity care, women with disabilities, inclusive healthcare, Africa

## Abstract

Women with disabilities face significant barriers in accessing maternal healthcare, which increases their risk of adverse pregnancy outcomes, particularly in Africa, where resources are limited. Artificial intelligence (AI) presents a unique opportunity to improve inclusivity and accessibility to antenatal care, skilled birth attendance and postnatal care for these women. This paper explores the potential of AI to address the socio-economic, physical, and institutional barriers that limit the utilisation of maternal healthcare services by women with disabilities. AI-driven technologies, such as virtual assistants, predictive analytics, and wearable devices, can enhance maternal health outcomes by improving monitoring during pregnancy, providing real-time health data, and facilitating access to skilled care. However, the successful implementation of AI in maternal healthcare in Africa faces challenges, including technological infrastructure, data quality, and ethical concerns. Collaborative efforts between governments, healthcare providers, and AI developers are necessary to overcome these challenges and ensure AI tools are inclusive, culturally sensitive, and accessible. Integrating AI into maternal healthcare services could lead to improved maternal outcomes, reduce mortality rates, and promote equity for women with disabilities in Africa.

## Background

Navigating pregnancy and childbirth, though fulfilling, is often complex and sometimes risky, particularly among vulnerable groups such as women with disabilities. Women with disabilities constitute a significant proportion of the global population, with one in every five women worldwide experiencing some form of disability,^1^ yet their maternal health issues remain largely ignored. Whilst all pregnant women are exposed to some level of adverse maternal outcomes, those with disabilities have a greater risk,^2^ especially in Africa, where there are limited resources and healthcare support systems for persons with disabilities. Meanwhile, sub-Saharan Africa alone accounts for about 70% of global maternal deaths,^3^ which occur mainly from preventable causes such as obstructed labour, hypertension, haemorrhage, and unsafe abortions.^4^ Although the exact prevalence of maternal mortality among women with disabilities is currently unknown, evidence suggests that women with disabilities have a higher likelihood of maternal deaths than those without disabilities.^2^ Meanwhile, improving access to maternal healthcare services such as antenatal care (ANC), skilled birth attendance (SBA), and postnatal care (PNC) could significantly reduce maternal morbidity and mortality in Africa, especially among women with disabilities.^2^

Over the past decade, the use of artificial intelligence (AI) has increased tremendously in various sectors, including healthcare, due to advancements in technology and machine learning.^5^ Whilst the use of AI in healthcare has shown great potential to improve access and utilisation of healthcare services,^5^ especially among vulnerable populations,^6^ its application and potential benefits in maternal healthcare for women with disabilities in Africa remain largely unexplored. With recent estimates showing stagnation in the decline of maternal mortality rates in most parts of Africa,^3^ there is a need for more innovative approaches, such as an integration of AI-driven technologies into maternal healthcare delivery to enhance access to ANC, SBA, and PNC, especially among disabled women who have increased limitations in accessing maternal healthcare services. Therefore, this commentary aims to highlight the potential of AI in maternal healthcare and the need to leverage AI technology to enhance inclusivity in the access and utilisation of maternal healthcare services among women with disabilities in Africa.

## Challenges faced by mothers with disabilities in maternity care

Women with disabilities encounter several socio-economic, physical, institutional, and cultural barriers which limit their ability to access or utilise maternal healthcare services, including ANC, SBA, and PNC. For instance, socio-economic challenges such as poverty, low literacy, and unemployment have been highly associated with limited access and utilisation of maternal healthcare services among women with disabilities in Africa.^3^

A study conducted by Ganle et al.^7^ reported that the high cost of transportation coupled with the lack of suitable means of transport and the distant location of healthcare facilities in many settings in Africa often make it difficult for many women with disabilities to access maternal healthcare services. Other barriers include a lack of physically accessible or disability-friendly healthcare facilities, limited ability to communicate with healthcare providers, and non-availability of institutional policies on healthcare for the disabled population.^7^

For example, Mitra et al.^8^ found that a lack of disability-specific clinical guidelines and information often poses difficulties to clinicians when providing maternity care to women with disabilities. Besides, poor health worker attitudes have also featured prominently as a major barrier to accessing maternal healthcare among disabled women,^7^ with evidence showing that many women with disabilities perceive their healthcare providers to possess negative views in relation to their sexuality, sexual activity, and childbearing.^8^

## How Artificial Intelligence can improve inclusivity in ANC

Although regular monitoring of pregnancy during the antenatal period is important in reducing pregnancy and childbirth-related complications, disabled women have increased limitations in access to ANC and other maternal healthcare services.^7^ With the ability to participate in human-like interactive conversations,^9^ AI-driven technologies such as ChatGPT can provide virtual assistants and personalised pregnancy-related health education and information,^10^ especially to disabled women with mobility and communication challenges.

Mugoye et al.^11^ proposed new AI-driven chatbots embedded in smart devices such as smartphones, which provide virtual health expertise and respond to queries from pregnant women in real time, which minimises the need to travel for ANC consultations. Besides, AI-driven m-health applications have been designed to promote consultations to facilitate client assessment and monitoring, particularly in promoted areas,^7^ thereby bridging the gap between women with disabilities and healthcare providers. Also, AI-driven machine learning and predictive analytics have been used to predict high-risk pregnancies,^12^ allowing tailored interventions for pregnant women with disabilities and enhancing pregnancy outcomes. Thus, the integration of AI applications in maternity care for women with disabilities could promote inclusivity and easy access to ANC, enhance monitoring and support, and predict risk factors during pregnancy, thereby enhancing maternal and child outcomes among women with disabilities in Africa.

## Artificial Intelligence-driven solutions for enhancing health facility deliveries and skill birth attendance

All pregnant women require SBA to reduce the risk of maternal and newborn deaths during delivery. However, the limitations of women with disabilities heighten their difficulties in accessing skilled delivery in many parts of Africa.^13^ AI applications can be used in the form of home monitors to provide real-time surveillance of pregnant women with disabilities and aid in clinical decision-making,^14^ including timely referrals to healthcare facilities. Also, AI applications can be used to optimise facility layouts and processes, ensuring accessibility and ease of navigation for women with disabilities.

For instance, AI-driven technologies such as route planning software and robotic arms ^14^ could aid in the mobility of pregnant women with disabilities to healthcare facilities during labour and thus promote health facility deliveries and ensure SBA. Whilst partographs are commonly used to monitor labour progress and guide decision-making during deliveries, the tool is often used inconsistently or incorrectly by healthcare workers in many settings, particularly in Africa.^13^

## Artificial Intelligence role in PNC for mothers with disabilities

Monitoring of mothers and their newborn babies during the postnatal period is essential in the early detection of post-delivery-related complications. Although there are limited applications of AI technology in PNC delivery,^15^ evidence suggests that AI technologies could potentially improve the monitoring of mothers and their newborn babies and aid in the early diagnosis of health risks and complications.^15^ With limited capabilities of women with disabilities often making identification of maternal and newborn health problems difficult after delivery,^15^ recent trials have shown that wearable devices powered by AI technology can provide remote monitoring of women’s postpartum health, including their mental health and physical recovery.^16^ Also, AI-enabled health tracking devices can be used to detect complications in newborns,^16^ especially in mothers with disabilities who may not be able to detect such anomalies on their own due to their disability. Further, virtual platforms can be used to provide mothers with disabilities access to PNC guidance,^10^ including breastfeeding, baby care, and maternal well-being.

## Addressing technological and infrastructural barriers to AI adoption in Africa

Despite having the capacity to revolutionise healthcare, particularly among disadvantaged populations such as women with disabilities, the adoption of AI in healthcare has largely remained slower than expected,^17^ especially in Africa. Several technological and infrastructural barriers impede the integration of AI technologies into healthcare delivery in Africa. First, AI applications operate on data sources, and thus, AI algorithms are as good as their data sources.^17^ However, aside from the scarcity of healthcare data, particularly maternal healthcare data of women with disabilities in Africa,^18^ most of the available datasets have limited quality and accuracy. The limited availability of maternal data on women with disabilities could hinder the development and training of disability-inclusive AI algorithms in maternal healthcare since using small datasets to validate AI could lead to inaccurate and misleading outputs.^17^ Therefore, aside from promoting the digitisation of healthcare data, there is a need for improved data collection on women with disabilities to enhance the development of targeted AI applications that promote effective maternity care for women with disabilities in Africa.

Also, the hardware infrastructure and IT systems required to develop and integrate AI technologies in healthcare are capital-intensive.^18^ Thus, considering the limited resources and competing demands for other healthcare expenditures in Africa, it is difficult to allocate enough resources to develop AI applications to promote access to maternity services among women with disabilities.

## Training healthcare providers for inclusive Artificial Intelligence-driven maternity services

Evidence suggests that many healthcare workers in Africa continue to have limited technological skills and capacity to operate AI-driven applications in healthcare.^19^ With the increasing momentum in the use of AI technologies in healthcare, it is envisaged that the future role of healthcare providers will be affected significantly, highlighting the need to educate healthcare workers on the importance of improving their technological skills and adjusting to working alongside AI technologies in healthcare delivery.^19^ Therefore, to successfully integrate AI solutions and improve access and utilisation of maternity services among women with disabilities in Africa, there is a need for enhanced AI literacy training and disability awareness creation among healthcare professionals.

## Ensuring ethical and culturally appropriate use of Artificial Intelligence in inclusive maternity care

AI algorithms require extensive datasets for training and ongoing validation or fine-tuning of their outputs.^20^ This reliance on data raises significant ethical concerns, particularly around issues such as data privacy, ownership, and consent. Protecting the privacy and data security of women with disabilities is vital for fostering trust in AI technologies and improving maternal health outcomes within this vulnerable population.^10,20^

However, it is also essential to consider the potential downsides of AI in inclusive maternity care. Two major challenges are information bias and algorithm bias. AI systems are only as reliable as the datasets on which they are trained. If these datasets are incomplete, biased, or fail to reflect diverse populations, the resulting algorithms may inadvertently exacerbate inequalities in maternity care.^20^ For instance, biases in the training data may lead to inaccurate or unsuitable recommendations for women with disabilities, particularly in settings with limited resources where cultural subtleties and specific needs are often underrepresented.^10^

A recent qualitative study assessing responses from ChatGPT to pregnancy-related queries found that only half of the information provided was accurate, whilst the rest was either inaccurate or incomplete.^20^ This underscores the risk of misinformation posed by AI tools, particularly in sensitive areas such as maternity care. Women with disabilities, who may have limited ability to critically assess the validity of AI-generated information, are especially vulnerable to the consequences of such inaccuracies.

Despite these challenges, the increasing reliance on AI tools such as ChatGPT by pregnant women seeking information about pregnancy-related concerns highlights the urgency of tailoring these technologies for use by women with disabilities.^10^ Targeted efforts are needed to enhance the reliability and usability of these tools whilst minimising the risks associated with biased or incorrect outputs.^10^ Addressing these issues requires incorporating diverse datasets that reflect the unique circumstances of disabled women and implementing safeguards to identify and mitigate inappropriate feedback.

By tackling these ethical and technical challenges, AI technologies can be refined to provide more accurate, culturally appropriate, and unbiased support for inclusive maternity care, ultimately improving outcomes for women with disabilities.

## Conclusion

AI-driven technologies have tremendous potential to transform maternal care and promote the inclusivity of women with disabilities in accessing and utilising ANC, SBA, and PNC. However, there is a need for collaborative efforts to ensure proper evaluation of AI-driven technologies in maternal healthcare for women with disabilities before they are widely adopted. Such collaborative efforts are required among policymakers, healthcare providers, AI developers, educational institutions, and disability advocacy groups to make maternity care more accessible and equitable whilst minimising the potential barriers and ethical issues associated with AI integration in maternal care for women with disabilities. This could potentially enhance access, improve maternal outcomes, and reduce maternal deaths, particularly among women with disabilities in Africa.

## Declarations

**Ethical approval and consent to participate:** Not applicable.

**Consent for publication:** Not applicable

**Availability of data and materials:** Not applicable.
